# Supplementary material for: Resolving the full spectrum of human genome variation using Linked-Reads
Source: Genome Res. 2019 Apr;29(4):635–45. doi: 10.1101/gr.234443.118 (PMC6442396; doi:10.1101/gr.234443.118)
Supplement: Supplemental Material [file supp_gr.234443.118_Supplemental_longranger-2.2.zip › longranger-2.2.tar.bz2!/lr2.2-public/lib/stan/cmdstan/stan/doc/api/html/structstan_1_1gm_1_1expression__visgen.html]

Stan: stan::gm::expression\_visgen Struct Reference


|  |  |
| --- | --- |
|  | Stan  2.5.0  probability, sampling & optimization |


- Main Page
- Related Pages
- Namespaces
- Classes
- Files

- Class List
- Class Index
- Class Hierarchy
- Class Members

All Classes Namespaces Files Functions Variables Typedefs Enumerations Enumerator Friends Macros Pages

- stan
- gm
- expression\_visgen

Public Member Functions |
List of all members

stan::gm::expression\_visgen Struct Reference

`#include <generator.hpp>`

Inheritance diagram for stan::gm::expression\_visgen:

|  |  |
| --- | --- |
| Public Member Functions | |
|  | expression\_visgen (std::ostream &o) |
|  | |
| void | operator() (nil const &) const |
|  | |
| void | operator() (const int\_literal &n) const |
|  | |
| void | operator() (const double\_literal &x) const |
|  | |
| void | operator() (const array\_literal &x) const |
|  | |
| void | operator() (const variable &v) const |
|  | |
| void | operator() (int n) const |
|  | |
| void | operator() (double x) const |
|  | |
| void | operator() (const std::string &x) const |
|  | |
| void | operator() (const index\_op &x) const |
|  | |
| void | operator() (const integrate\_ode &fx) const |
|  | |
| void | operator() (const fun &fx) const |
|  | |
| void | operator() (const binary\_op &expr) const |
|  | |
| void | operator() (const unary\_op &expr) const |
|  | |
| Public Member Functions inherited from stan::gm::visgen | |
|  | visgen (std::ostream &o) |
|  | |

|  |  |
| --- | --- |
| Additional Inherited Members | |
| Public Types inherited from stan::gm::visgen | |
| typedef void | result\_type |
|  | |
| Public Attributes inherited from stan::gm::visgen | |
| std::ostream & | o\_ |
|  | |

## Detailed Description

Definition at line 138 of file generator.hpp.

## Constructor & Destructor Documentation

|  |  |  |  |  |  |
| --- | --- | --- | --- | --- | --- |
| stan::gm::expression\_visgen::expression\_visgen | ( | std::ostream & | *o* | ) |  |

inline

Definition at line 139 of file generator.hpp.

## Member Function Documentation

|  |  |  |  |  |  |
| --- | --- | --- | --- | --- | --- |
| void stan::gm::expression\_visgen::operator() | ( | nil const & |  | ) | const |

inline

Definition at line 140 of file generator.hpp.

|  |  |  |  |  |  |
| --- | --- | --- | --- | --- | --- |
| void stan::gm::expression\_visgen::operator() | ( | const int\_literal & | *n* | ) | const |

inline

Definition at line 143 of file generator.hpp.

|  |  |  |  |  |  |
| --- | --- | --- | --- | --- | --- |
| void stan::gm::expression\_visgen::operator() | ( | const double\_literal & | *x* | ) | const |

inline

Definition at line 144 of file generator.hpp.

|  |  |  |  |  |  |
| --- | --- | --- | --- | --- | --- |
| void stan::gm::expression\_visgen::operator() | ( | const array\_literal & | *x* | ) | const |

inline

Definition at line 150 of file generator.hpp.

|  |  |  |  |  |  |
| --- | --- | --- | --- | --- | --- |
| void stan::gm::expression\_visgen::operator() | ( | const variable & | *v* | ) | const |

inline

Definition at line 164 of file generator.hpp.

|  |  |  |  |  |  |
| --- | --- | --- | --- | --- | --- |
| void stan::gm::expression\_visgen::operator() | ( | int | *n* | ) | const |

inline

Definition at line 165 of file generator.hpp.

|  |  |  |  |  |  |
| --- | --- | --- | --- | --- | --- |
| void stan::gm::expression\_visgen::operator() | ( | double | *x* | ) | const |

inline

Definition at line 166 of file generator.hpp.

|  |  |  |  |  |  |
| --- | --- | --- | --- | --- | --- |
| void stan::gm::expression\_visgen::operator() | ( | const std::string & | *x* | ) | const |

inline

Definition at line 167 of file generator.hpp.

|  |  |  |  |  |  |
| --- | --- | --- | --- | --- | --- |
| void stan::gm::expression\_visgen::operator() | ( | const index\_op & | *x* | ) | const |

inline

Definition at line 168 of file generator.hpp.

|  |  |  |  |  |  |
| --- | --- | --- | --- | --- | --- |
| void stan::gm::expression\_visgen::operator() | ( | const integrate\_ode & | *fx* | ) | const |

inline

Definition at line 180 of file generator.hpp.

|  |  |  |  |  |  |
| --- | --- | --- | --- | --- | --- |
| void stan::gm::expression\_visgen::operator() | ( | const fun & | *fx* | ) | const |

inline

Definition at line 203 of file generator.hpp.

|  |  |  |  |  |  |
| --- | --- | --- | --- | --- | --- |
| void stan::gm::expression\_visgen::operator() | ( | const binary\_op & | *expr* | ) | const |

inline

Definition at line 235 of file generator.hpp.

|  |  |  |  |  |  |
| --- | --- | --- | --- | --- | --- |
| void stan::gm::expression\_visgen::operator() | ( | const unary\_op & | *expr* | ) | const |

inline

Definition at line 242 of file generator.hpp.

---

The documentation for this struct was generated from the following file:

- src/stan/gm/generator.hpp


---

[ Stan Home Page ]

*© 2011–2014,
Stan Development Team.*
